# Optimization of Ultrasound-Assisted Extraction of Chlorogenic Acid from Potato Sprout Waste and Enhancement of the In Vitro Total Antioxidant Capacity

**DOI:** 10.3390/antiox12020348

**Published:** 2023-02-01

**Authors:** Luciano Mangiapelo, Francesca Blasi, Federica Ianni, Carolina Barola, Roberta Galarini, Ghaid WA Abualzulof, Roccaldo Sardella, Claudia Volpi, Lina Cossignani

**Affiliations:** 1 Department of Pharmaceutical Sciences, University of Perugia, Via Fabretti 48, 06123 Perugia, Italy; 2 Istituto Zooprofilattico Sperimentale dell’Umbria e delle Marche “Togo Rosati”, 06126 Perugia, Italy; 3 Center for Perinatal and Reproductive Medicine, Santa Maria della Misericordia University Hospital, University of Perugia, Sant’Andrea delle Fratte, 06132 Perugia, Italy; 4 Department of Medicine and Surgery, University of Perugia, Piazzale Gambuli 1, 06132 Perugia, Italy

**Keywords:** potato sprout waste, UAE, antioxidant activity, chlorogenic acids, Liquid Chromatography, enzymatic browning, Experimental Design

## Abstract

Potato sprouts, an underutilized by-product of potato processing, could be exploited for the recovery of caffeoyl-quinic acids (CQAs), a family of polyphenols with well-recognized biological activities. In this work, the predominant compound of this class, 5-CQA, was extracted by Ultrasound-Assisted Extraction (UAE) under conditions optimized by an Experimental Design. The investigated variables solid/solvent ratio (1:10–1:50 g/mL), water content in ethanol (30–100% *v*/*v*) and UAE time (5–20 min) highlighted a critical influence of the last two factors on the extraction efficiency: extracts richer in 5-CQA were obtained with lower water content (30%) and time (5 min). The addition of ascorbic acid (1.7 mM) as anti-browning agent to the extraction solvent improved the extraction efficiency of 5-CQA compared to acetic and citric acids (3158.71 μg/mL, 1766.71 μg/mL, 1468.20 μg/mL, respectively). A parallel trend for the three acids and an increase in 5-CQA recovery was obtained with the use of freeze-dried sprouts (4980.05 μg/mL, 4795.62, 4211.25 μg/mL, respectively). Total antioxidant capacity (TAC) in vitro demonstrated UAE being a more valuable technique than conventional maceration. Furthermore, three-times-higher values of TPC (7.89 mg GAE/g) and TAC (FRAP: 24.01 mg TE/g; DPPH: 26.20 mg TE/g; ABTS 26.72 mg TE/g) were measured for the optimized extract compared to the initial one. An HPLC-DAD method was applied to monitor 5-CQA recovery, while an LC-HRMS/MS investigation allowed us to perform analyte identity confirmation along with detection of the glycoalkaloids α-solanine and α-chaconine. This evidence underlines the necessity to develop purification strategies in order to maximize the potential of potato sprout waste as a source of 5-CQA.

## 1. Introduction

Potato (*Solanum tuberosum*) tubers have emerged in the commercial panorama as one of the most important food crops worldwide. This humble tuber, historically recognized as a starchy food, has been a staple of the diet for generations for many hundreds of years. Despite the high popularity, potatoes have managed to stir up some controversy, and their impact on human health has sometimes been the object of prejudice [1]. On the other hand, potatoes are recognized to play an important role in human health and malnutrition prevention. In fact, apart from the above negative connotation, potatoes are a valuable source of important nutrients including vitamins and micronutrients, dietary fiber, and several types of phytonutrients as carotenoids, anthocyanins, chlorogenic and caffeic acids, which promote such tuber as healthy food.

Likewise, the by-products of potato processing, consisting of roots, leaves, peels, and sprouts, offer a wide range of functional compounds with high nutritional value. In this frame, the growing attention towards the valorization of food waste also involves potato processing industries, which are responsible for the generation of a huge volume of wastes and by-products. Through recycling actions, problems related to waste disposal, environmental pollution and sanitation concerns can be sensitively mitigated [2,3]. However, many bioactive compounds, in particular phenolic acids and other trace elements and vitamins, are located in the peel and adjacent tissues and eliminated as waste. For this reason, numerous efforts are still being made to improve extraction protocols and technology-aided approaches for the recovery of valuable compounds from potato by-products, in more sustainable, energy-efficient, and cost-effective ways [4,5]. In this context, potato peel stands out as the major by-product obtained from potato processing and is studied as a source of phytochemicals with potential pharmacological properties that could contribute to the development of novel and healthy functional foods [6,7]. Chlorogenic acid (5-O-caffeoyl-quinic acid, 5-CQA), its isomers neochlorogenic (3-CQA) and cryptochlorogenic (4-CGA) and caffeic acid, are among the predominant bioactive compounds found in potato peels [2,6,7]. These dietary polyphenols are recognized to possess several outstanding pro-health properties including antioxidant, radical scavenging, anticancer, antiobesity, and antidiabetic activity [8,9]. Other potato processing rejects include inedible sprouts, which have gained only marginal attention so far, even though they are a rich source of CQAs and other phenolic compounds [10,11,12]. Their unpopularity relates to the presence of toxic steroidal glycoalkaloids (GAs), α-solanine (solanidine-galactose-glucose-rhamnose) and α-chaconine (solanidine-glucose-rhamnose-rhamnose). More specifically, potato sprouts and flowers contain the highest level of GAs followed by peels, and finally fresh tubers [13]. Their increased content in turned-green and sprouting or rotting potatoes dramatically decreases the commercial value, making them unusable for human consumption if the recommended limit (200 mg/kg fresh weight, corresponding to 1000 mg/kg dry weight) is exceeded [14]. In spite of their toxic effects [13], recent studies have highlighted beneficial properties of α-solanine and α-chaconine including anti-inflammatory, antitumor and antibiotic activity against pathogens, depending on concentration and condition of use [15,16,17,18,19].

The above evidence highlight a double-edged sword role played by potato sprouts: on the one hand, they are a valuable resource of phytochemicals including phenols with antioxidant properties; on the other, they are a source of cytotoxic GA constituents, with potential medicinal implication [18,19,20]. Earlier studies [11,21] have highlighted a high concentration of 5-CQA in the sprouts of seven varieties of potatoes (ranging from 10 to 19 mg/100 g of fresh weight) and the highest content in sprouts of an experimental potato plant (754 mg/100 g of fresh weight) followed by leaves (224 mg/100 g), roots (26 mg/100 g), and finally tubers (17 mg/100 g). It is noteworthy that this content was roughly paralleled by the GA content, reflecting the high metabolic rate of sprouts with the consequent activation of pathways involved in their biosynthesis [22,23].

Due to the biological relevance of 5-CQA, the aim of this study was to identify the active constituents of different water-based extracts of potato sprouts obtained by Ultrasound-Assisted Extraction (UAE). With the aim of obtaining an end product with a high content of chlorogenic acid and derivatives, a comparative evaluation of different experimental conditions was performed to select the most appropriate. The optimization of UAE conditions was performed and monitored via reversed-phase HPLC-DAD analysis, after a preliminary comparison with the traditionally applied Maceration Extraction (ME) [24]. The influence of critical variables on the efficiency of the UAE-based extraction process was evaluated in depth by an Experimental Design. Finally, LC-HRMS/MS analysis was performed on the optimal extract to confirm the chemical identity of CQAs, followed by the evaluation of the in vitro total antioxidant capacity.

To the best of our knowledge, this is the first study in which a systematic approach for the optimization of 5-CQA isolation from potato sprouts is described. The use of an underestimated waste as a source of relevant bioactive compounds further strengthens the novelty of our study.

## 2. Materials and Methods

### 2.1. Chemicals

All the employed solvents were of analytical grade and purchased from Carlo Erba (Milan, Italy). Chlorogenic acid (5-CQA) and neochlorogenic acid (3-CQA) standards were purchased from Sigma Aldrich (Milan, Italy), while cryptochlorogenic acid (4-CQA) and caffeic acid (CA) standards were purchased from PhytoLab (Vestenbergsgreuth, Germany). Acetonitrile (ACN) and methanol, both LC-MS grade, were from Merck KGaA (Darmstadt, Germany). Formic acid for LC-MS was purchased from Sigma-Aldrich (St. Louis, MO, USA). Folin–Ciocalteu reagent, 2,2′-azino-bis-(3-ethylbenzothiazoline-6-sulphonate) diammonium salt (ABTS), 2,4,6-tris(2-pyridyl)-s-triazine (TPTZ), 6-hydroxy-2,5,7,8-tetramethyl-2-carboxylic acid (Trolox), 2,2-diphenyl-1-picrylhydrazyl (DPPH), ferric chloride (FeCl_3_), and gallic acid (GA) were purchased from Sigma-Aldrich (Milan, Italy). Ultra-pure deionized water was generated by a Milli-Q purification apparatus (Millipore, Bedford, MA, USA).

### 2.2. Plant material

Potato samples were provided by a local farmer in Balanzano (Province of Perugia, Umbria Region, Italy). Three different sprouting stages were evaluated:

(i) Stage-1: sprouts already present on the purchased potatoes;
(ii) Stage-2: sprouts from potatoes left sprouting for two months after the collection of stage-1 sprouts in a humid and lighted environment;
(iii) Stage-3: sprouts from potatoes left sprouting for three months after the collection of stage-2 sprouts in a humid and lighted environment.

In all cases, sprouts (fresh and oven-dried) were weighed and treated with the selected extraction solvent and then homogenized using a three-blade blender (Osterizer, Oster, model 869-50R, Milwaukee, WI, USA) for 2 min. Freeze-dried samples did not re-quire the homogenization step.

### 2.3. Maceration Extraction (ME) of CQAs from Potato Sprouts

Fresh potato stage-1 sprouts were extracted by maceration to select the most per-forming extraction technique between conventional and non-conventional approaches. Sprouts were treated with the selected extraction solvent acetone/water ratio of 70:30 (*v*/*v*) containing 1.7 mM acetic acid (Hac) [12] in a solid/liquid ratio of 1:60 (*w*/*v*). The homogenized mixture was kept under stirring for 4 h, at room temperature [25]. After ME, the extracts were centrifuged (Neya 8-REMI, Carpi, Italy) at 4500 rpm for 5 min, at room temperature, and the supernatants were filtered.

### 2.4. Ultrasound-Assisted Extraction (UAE) of CQAs from Potato Sprouts

UAE was performed for 5 min (sonicator bath Model AU-65, by ARGOLAB, Carpi, Italy) with the selected extraction solvent as follows:

(i) Stage-1 sprouts were used for the preliminary optimization of the extraction method. The phenolic profile of fresh extracts was compared with that of oven-dried sprouts, at 40 °C (for 5 h and 30 min) and 70 °C (for 4 h). UAE was carried out for 5 min by using a mixture of acetone/water (70:30, *v*/*v*) containing 1.7 mM acetic acid (HAc) [12]. Ethanol/water (70:30, *v*/*v*) and pure water, in both cases containing HAc 1.7 mM, were also compared. Stage-1 sprouts were also used for the optimization of the UAE conditions by Experimental Design (see Section 2.5);
(ii) Stage-2 sprouts were submitted to the optimal UAE conditions as defined by the Experimental Design. Different acid additives, citric acid (CitA) and ascorbic acid (AsA) were evaluated as anti-browning agents. The comparison between fresh and freeze-dried sprouts was also investigated;
(iii) Stage-3 sprouts were submitted to the best identified extraction conditions.

Homogenized samples were vortexed (model ZX3 advanced vortex mixer, VELP Scientifica Srl, Usmate, Italy) within a timeframe of 30–60 s and subjected to UAE. The obtained extracts were centrifuged at 4500 rpm for 5 min, at room temperature, and the supernatants were filtered using a syringe filter (nylon membrane 25 mm, 0.45 μm pore size, VWR international, Milan, Italy).

### 2.5. Experimental Design

The optimization of the extraction conditions of CQAs from potato sprouts was achieved by applying an Experimental Design, using MODDE 5.0 (UMETRICS AB, Umeå, Sweden) software.

The following quantitative factors were considered:

- Water content (% volume in ethanol), ranging between 30% and 100%;
- Solid/solvent ratio (g/mL), ranging between 1:10 and 1:50;
- UAE time (min), ranging between 5 min and 20 min.

**Scheme 1:**
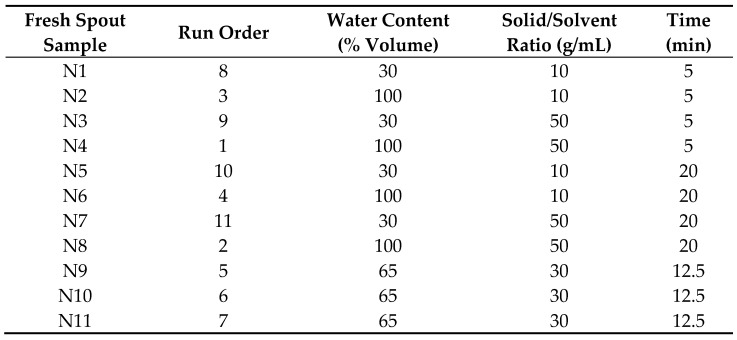
Experimental design: independent variables for UAE experiments.

The influence of these factors was investigated on the 5-CQA content as response. A full factorial design (two levels) was selected, indicating a total of 11 experiments including 3 replicated center points (N 1–11). The extractions were carried out in random order using the experimental conditions indicated in the worksheet by the software (Scheme 1). The model was then obtained using Partial Least Squares (PLS) regression analysis.

### 2.6. Total Phenol Content (TPC) and In Vitro Antioxidant Activity

All the assays were performed according to our previous papers [26,27] with slight modifications.

TPC was determined using the Folin–Ciocalteu method. The phenolic content of the extract solutions was determined by measuring the absorbance at 765 nm. The value was quantified using a calibration curve of gallic acid and results were expressed as mg gallic acid equivalents per gram of dry potato sprouts (mg GAE/g).

The reducing capacity was evaluated using the FRAP assay and measuring the absorbance at 593 nm after 30 min incubation of the extract with the Fe^3+^-TPTZ complex to achieve the reduction. The free radical-scavenging activity using DPPH and ABTS assays. The DPPH reagent was added to the extract sample and the absorbance measured at 517 nm, after 30 min incubation of the mixture. ABTS reagent was prepared and added to the extracts. The absorbance was measured at 734 nm after keeping the mixture in the dark for 6 min.

Quantification for FRAP, DPPH and ABTS assays was performed via a calibration curve of Trolox, and the results were expressed as mg of Trolox equivalent per gram of dry potato sprout (mg TE/g). All the UV spectra were recorded with a Sunrise microplate reader (Tecan Srl, Milan, Italy) using a disposable optical Corning^®^ 96-well plates (Merck Life Science, Darmstadt, Germany).

### 2.7. HPLC-DAD Analysis and Method Validation

A previously optimized HPLC-DAD method [26] was applied to evaluate the qualitative and quantitative profile of 5-CQA and related compounds. The HPLC measurements were made on a Thermo Separation low-pressure quaternary gradient pump system coupled to a Spectra system UV 6000 LP diode array detector (DAD) (Thermo Scientific, Waltham, MA, USA), supplied with a GT-154 vacuum degasser (Shimadzu, Kyoto, Japan), and a Rheodyne7725i injector (Rheodyne Inc., Cotati, CA, USA) with a 20 μL stainless steel loop. Data acquisition was performed by the Excalibur software (Chromatographic Specialties Inc., Brockville, Canada). A HyperSil GOLD™ C18 column (150 × 4.6 mm, 3 μm particle size, 175 Å pore size, by Thermo Fisher Scientific, Waltham, MA, USA) was used as stationary phase. The column was conditioned for 20 min before use with the selected mobile phase. The isocratic mode of elution based on the use of water (containing 0.1% formic acid) and acetonitrile (containing 0.1% formic acid) (97:3, *v*/*v*). The employed mobile phase components were degassed by sonication for 15 min before use and flowed through the column at a 1.0 mL/min flow rate. The detection of 5-CQA and its isomers (3-CQA and 4-CQA) as well as of caffeic acid (CA) was monitored at a wavelength of 325 nm.

Calibration curves by using standard solutions of CA, 3-CQA, 4-CQA and 5-CQA were built up to determine the concentration of the analytes in the extracts. The good linearity of the calibration curves is expressed by the high R^2^ values (>0.998) obtained (Table S1, Supplementary Material) for all the investigated species. The reliability of the HPLC method was established through a basic “research” level validation, in terms of accuracy and precision, limit of detection (LOD) and limit of quantification (LOQ) (Tables S1 and S2, Supplementary Material), with the use of two different control solutions with theoretical concentrations fixed at 3.65 and 14.60 µg/mL, respectively. High recovery% values and a low range of variation of the RSD% values (<3) were observed for the evaluation of the short-(intra-day) and long-term (inter-day) accuracy and precision, respectively. In addition, very low LOD and LOQ values were estimated for all the investigated analytes as shown in Table S2 (Supplementary Material). The results demonstrated that the developed method was reliable and suitable for quantitative analysis to be performed.

### 2.8. LC-HRMS/MS Analysis

Chromatography was performed on a Thermo Ultimate 3000 High Performance Liquid Chromatography system (Thermo Scientific, San Jose, CA, USA). Analytes were separated on a Xbrigde BEH C18 column (100 × 2.1 mm, 2.5 µm, Waters, Milford, MA, USA). In both ionization modes LC eluent “A” was an aqueous solution containing 0.1% (*v*/*v*) formic acid and eluent “B” was acetonitrile. The gradient was initiated with 100% eluent “A” at 0.3 mL/min. Eluent “B” increased to 15% in 15 min, the gradient continued with an increase to 100% “B” in 3 min. This condition was maintained for 2.5 min. Then, the system returned to 100% “A” in 0.5 min. The re-equilibration was 3 min. The column temperature was 30 °C, the autosampler temperature was kept at 10 °C and injection volume was 5 µL.

The mass spectrometer Q-Orbitrap (Thermo Scientific) was equipped with heated electrospray ionization (HESI-II) source. The HESI-II and capillary temperatures were set at 320 °C and 300 °C, respectively, and the electrospray voltage at 3.50 kV and −3.50 kV in positive and negative ionization mode, respectively. Sheath and auxiliary gas were 35 and 15 arbitrary units. The acquisition was achieved in full scan/dd-MS^2^. In negative ionization mode the mass range was *m*/*z* 100–1200, whereas in positive mode, the range was *m*/*z* 180–950. Resolving power was set at 35,000 FWHM (at *m*/*z* 200) in full MS mode. Automatic Gain Control (AGC) was set at 1 × 106 ions with a maximum injection time (IT) of 160 ms. A resolving power of 17500 FWHM (*m*/*z* 200) was used for the MS^2^ experiments with an AGC target of 1 × 106 ions for a maximum IT of 80 ms. Stepped collision energies at 15, 40 and 80 (NCE) were applied in both ionization modes. Two inclusion lists were built to perform full-MS/dd-MS^2^ experiments in positive and negative ionization mode, respectively, reporting the m/z of precursor ions and their expected retention times (±1 min), when authentic standards were available. MS analyzer parameters were the same both in ESI− and ESI+. Mass extraction window was 5 ppm (mass error).

### 2.9. Statistical Analysis

The results are expressed as mean value ± standard deviation (*n* = 3). Statistical significance was measured through one-way analysis of variance (ANOVA) followed by Tukey’s honestly significant difference post hoc. OriginPro 9.0 (OriginLab Corporation, Northampton, MA, USA) was used as statistical software. Values with *p* < 0.01 and *p* < 0.05 were considered significant.

## 3. Results and Discussion

### 3.1. Preliminary Extraction of Chlorogenic Acids: ME vs. UAE

**Figure 1:**
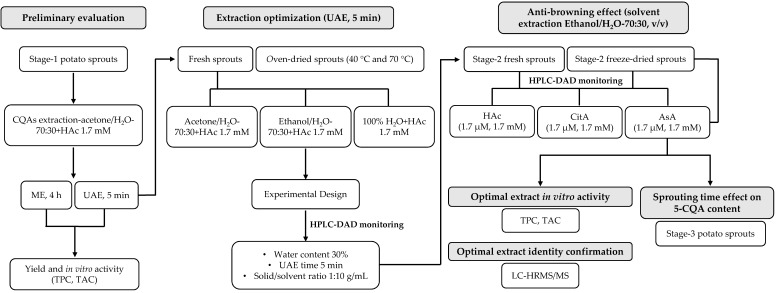
Overview of extraction optimization for 5-CQA recovery from potato sprouts.

In this study, UAE was applied to perform the extraction of 5-CQA and its isomers from potato sprouts and compared with ME. Although innovative technologies can produce higher yield and better quality of the extracts by applying milder conditions [28], traditional extractions are often used as reference methods to compare the success of a newly developed methodology [29,30]. The performance of both techniques was appraised by measuring the extraction yield, total phenol content (TPC) and bioactivity by in vitro antioxidant assays. An overview of the extraction process is shown in Figure 1.

For the preliminary comparison, the mixture acetone/water (70:30, *v*/*v*) containing 1.7 mM acetic acid (HAc) was used with UAE carried out for 5 min [12], while ME was protracted for 4 h [25]. At this early evaluation, the extraction was performed on stage-1 sprouts, emerging from potatoes at the time of purchase (see Section 2.2 for details). As evident from Table 1, generally higher values were obtained for UAE than ME, highlighting a faster and better extraction efficiency of the unconventional technique. As far as TPC, FRAP, and ABTS values are concerned, ANOVA analysis enlightened a high statistical difference (*p* < 0.01) between the results obtained with the two extraction techniques. A *p*-value lower than 0.05 was obtained for DPPH.

The substantial saving in terms of time and energy consumption with the proposed UAE method [31] is accompanied by a series of benefits related to the improved solvent penetration within the plant tissue and the intensification of the mass transfer process. This, in turn, reflects the higher TPC and TAC values of the UAE vs. ME extract (Table 1). The slightly higher ABTS response obtained with ME, plausibly correlated to unspecific reactions [32], is largely counterbalanced by the reduced extraction time achievable with UAE. Based on the above, the remarkable performances provided by UAE supported the selection of this non-conventional technique for subsequent experiments.

### 3.2. Optimization of Chlorogenic Acid Extraction by UAE: Fresh vs. Oven-Dried Potato Sprout Samples

In this stage of the study, the CQAs extraction from Stage-1 sprouts (see Section 2.4 for details) was performed by comparing fresh and oven-dried starting material. Dry matter content was determined for the two investigated temperatures after 5.5 h (40 °C) and 4 h (70 °C), respectively, until a constant weight was reached. In both cases, a water loss of approximately 70% was obtained. The mixture was submitted to UAE extraction for 5 min by using an acetone/water (70:30, *v*/*v*) solution containing 1.7 mM acetic acid (HAc). The HPLC-DAD analysis, optimized in a previous study [26], showed the presence of four main peaks identified as 5-CQA and its main isomers, neochlorogenic acid (3-CQA) and cryptochlorogenic acid (4-CQA), and caffeic acid (Figure S1A, Supplementary Material). The chromatographic profiles obtained for the fresh and oven-dried potato sprouts showed notable differences among the three extracts (Figure S1B–D, Supplementary Material). The quantitative results reported in Table 2 evidenced a different impact of temperature on 5-CQA stability. Low recovery of CA and 5-CQA and no traces of 3-CQA and 4-CQA were observed in the dried sprouts with respect to the fresh ones.

The well-known thermosensitivity of 5-CQA could explain the unfavorable effect of the temperature, responsible for triggering a degradation process that lasts over time [26,33].

In order to evaluate the extraction efficiency with the use of more environmentally friendly solvents than acetone, two further solvent systems based on ethanol/water (70:30, *v*/*v*) and pure water (100%), both added with HAc 1.7 mM, were compared on fresh potato sprouts. As reported in Table 3, the ethanol-based system afforded the best results, while the extraction with 100% water proved to be the weakest to properly extract the compounds of interest.

### 3.3. Optimization of UAE Extraction of CQAs by Experimental Design

Based on the obtained results, the Experimental Design software MODDE^®^ 5.0 (UMETRICS, Umeå, Sweden) was employed to evaluate the influence of three independent parameters (water content in ethanol, solid/solvent ratio, time) on the 5-CQA extraction from fresh potato sprouts by UAE [34]. However, all phenolic acids studied were quantified by HPLC-DAD in the indicated eleven experiments, and the response of the Experimental Design was evaluated in terms of 5-CQA yield (μg/g) (Table 4).

**Figure 2:**
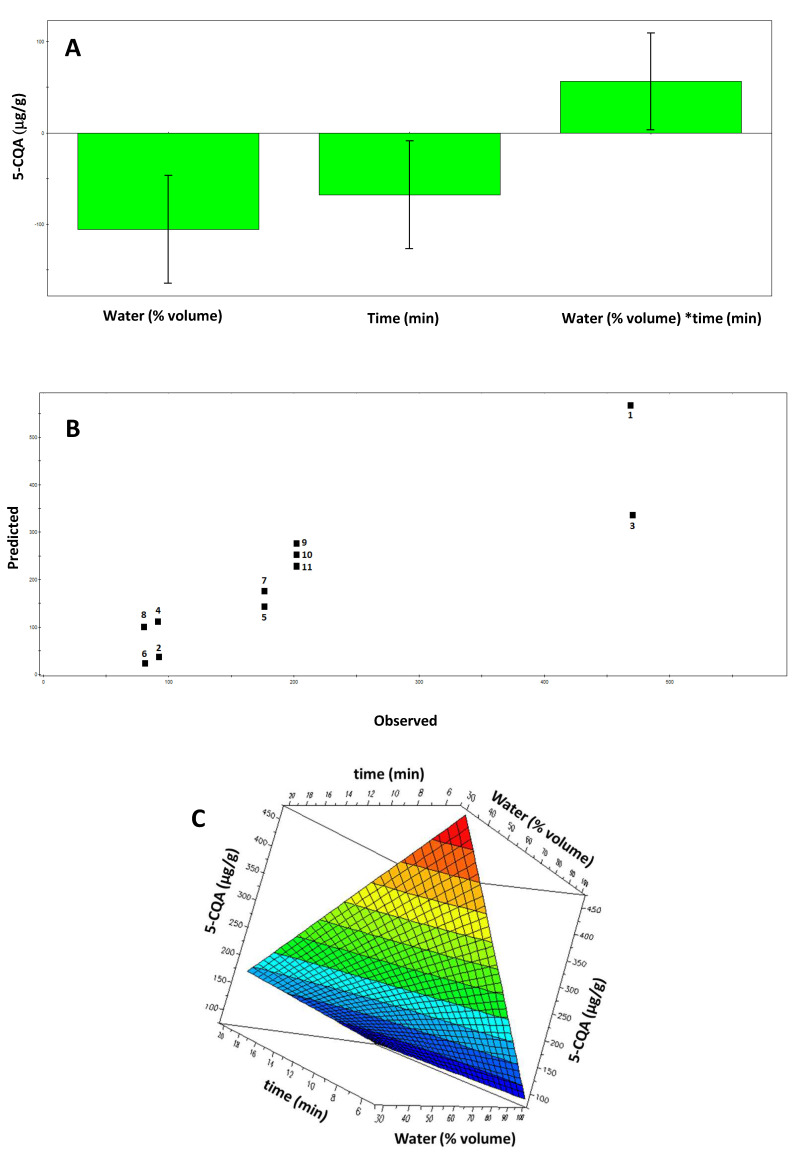
Results of the experimental design: (**A**) coefficient plot, (**B**) observed vs. predicted plot, (**C**) response surface.

The quality of the mathematical model obtained was evaluated by two statistical parameters, R^2^ (which describes how well the model fits the experimental data) and Q^2^ (which describes how well the model will predict new data). The satisfactory observed values (R^2^ = 0.818, Q^2^ = 0.534) indicated the goodness-of-fit of the statistical model and its appropriateness for optimization and prediction purposes. Figure 2A shows the coefficients of the selected factors and their interaction for 5-CQA response, that is, the influence of the extraction factors on the considered response. It is possible to observe that the factors solvent composition (water content in ethanol) and time influenced in a negative manner the response, with a generally higher effect for water content with respect to time. These results indicate that sprout extracts higher in phenolic acids can be obtained with the lowest values of water content (30%) in the hydroalcoholic mixture and in a shorter time (5 min). It should be emphasized that the solid/solvent ratio was removed from the model, due to its negligible influence on the response. This result shows that it is possible to adopt the lowest solid/solvent ratio to extract 5-CQA from sprouts without compromising the bioactive extraction yield. Interestingly, the low solvent consumption makes the extraction process cost-effective and environmentally friendly. Furthermore, the low extraction time makes the process faster and cheaper. In Figure 2B, the observed vs. predicted plot shows that the points are close to a straight line, with a little deviation of sample 3, demonstrating the good validity of the model. Lastly, Figure 2C shows the surface plot generated by the software, a graphical representation of the experimental region with 5-CQA response as a function of water content and time factors. It is evident that the decrease in water % of ethanol and time corresponds to an increase in the responses.

### 3.4. Evaluation of Anti-Browning Acid Additives

The optimized UAE conditions, as indicated by the Experimental Design, were applied to perform subsequent extractions of 5-CQA and related compounds. The influence of ascorbic acid (AsA) and citric acid (CitA) in place of HAc on extraction efficiency was assayed by using Stage-2 sprouts (see Section 2.4 for details). In fact, a mere visual evaluation of fresh-cut homogenized sprouts revealed a certain degree of browning likely attributable to the polyphenol oxidase activity that, in the presence of oxygen, converts phenolic compounds into dark colored pigments. The contribution of other enzymes such as peroxidase to total browning may also be relevant [35]. Both AsA and CitA are extensively used to avoid enzymatic browning of fruit and vegetables due to the reduction in the *o*-quinones, generated by the enzymes action on phenolic substrates. Additionally, an important advantage of these natural anti-browning agents is their designation as “generally recognized as safe” (GRAS) additives. The selected acid was added in the optimal extraction solvent before the homogenization step. The quantitative analysis reported in Table 5 shows the results obtained by using two different concentrations, 1.7 μM and 1.7 mM, for the three acids.

As a general observation, the use of each additive at a concentration of 1.7 mM proved to be more efficient in the extraction of all the investigated species. Moreover, when a low additive concentration was used, similar results have been obtained for HAc and AsA, which facilitated a higher extraction yield compared to CitA. A different trend was instead obtained when the concentration of the acid additive was 1.7 mM. In this case, in fact, the content of CQAs and CA was generally double by adding AsA to the extraction mixture, compared to the other additives. The obtained findings corroborated the effectiveness of AsA as natural agent over HAc and CitA in the control of the enzymatic browning in fresh-cut sprouts.

### 3.5. Fresh vs. Freeze-Dried Potato Sprout Samples

A freeze-drying procedure was applied as an additional strategy to prevent enzymatic browning and to ensure longer preservation of potato sprout samples. This process caused the ponderal loss of around 80% of water in sprouts and profitably avoided the browning process. Analogously to fresh sprouts, freeze-dried samples were monitored by HPLC after extraction with HAc, AsA and CitA at the two concentrations (Table 6).

As clearly evident, freeze-dried potato sprouts resulted in a higher extraction yield of 5-CQA and its isomers than fresh ones (*p* < 0.01). In conclusion, both lyophilization and AsA treatment resulted in a significant decrease in enzymatic browning, allowing an improved recovery of the examined compounds (Figure S2, Supplementary Material).

In order to evaluate the biological activities of the optimized extract [36], in vitro TAC was compared with the extract obtained in the starting operating conditions, previously shown in Table 1. A three-fold increase in TPC (7.89 ± 0.33 mg GAE/g) and TAC values (FRAP: 24.01 ± 1.82 mg TE/g; DPPH: 26.20 ± 0.72 mg TE/g; ABTS 26.72 ± 1.11 mg TE/g) was recorded with respect to the early conditions. Taking into consideration the above-mentioned results, this study clearly highlighted the pivotal role of extraction technique and optimization process to enhance the bioactivity of phenolic extracts from natural sources with promising antioxidant properties.

### 3.6. Evaluation of the Sprouting Time on 5-CQA Content

With the aim of achieving a preliminary assessment of the impact of sprouting time on the CQA content, HAc and AsA 1.7 mM were compared on Stage-3 sprouts (see Section 2.2 for details). A remarkable reduction (*p* < 0.01) in the content of CA, 5-CQA and 4-CQA was clearly highlighted by chromatographic results of these extracts (Table 6) compared with the previous ones (Stage-2 sprouts). Possible reasons for the observed depletion of such bioactive compounds with increasing sprouting time could likely be attributed to genetic mechanisms, and/or environmental factors, which control their distribution among the several parts of the tuber [10].

A controversial behavior, in this case, concerns the higher 5-CQA content resulting from the use of HAc compared to AsA. During post-harvest storage, polyphenol oxidase activity reflects the aging of potato tubers. In this stage, the evolution of physiological processes influences the sprouting pattern: the onset of tuber sprouting could trigger the polyphenol oxidase activities and cause a significant drop in the phenolic content [37,38,39]. Furthermore, a general increase in oxidative stress takes place during storage [37,40] in which the activation of other metabolic pathways during potato sprouting could not be ruled out. The overlap of all these factors could greatly influence the biochemical profile of sprouts, thus explaining the loss of effectiveness of AsA in helping to manage enzymatic activities.

### 3.7. Results of LC-HRMS/MS Analysis

The objective of the LC-HRMS investigation was the identity confirmation of the main peaks, that is, 5-CQA, followed by its isomers 3- and 4 CQA, and CA, obtained by UAE treatment of potato sprouts. The negative ionization mode applied on the optimal extract, established according to Section 2.4, confirmed the identity of CQA isomers as well as of CA (Figures S3 and S4, Supplementary Material). The study of the fragment ions has also led to the identification of minor polyphenol components (Figure S5 Supplementary Material).

High resolution mass spectrometry operating in positive ionization mode was applied to identify potato GAs; the ions at *m*/*z* 868.5025 and *m*/*z* 852.5091 were attributed to protonated α-solanine and α-chaconine molecules, respectively (Figures S6 and S7, Supplementary Material). The confirmation of both compounds was based on the comparison between the recorded fragment ions with data reported in the literature [41,42]. The simulation of the fragmentation processes using Mass Frontier^TM^ package (Thermo Scientific, San José, CA, USA) was performed to explain the observed fragment ions. MS^2^ results showed the cleavage sequence of the different sugar moieties [43]. The facile release of monosaccharide α-L-rhamnose generated the product ion at *m*/*z* 722.4453, while the ion at *m*/*z* 706.4518 can be attributed to the loss of β-D-glucose unit. The fragmentation pathways for these compounds proceeded with the loss of the last monosaccharide to produce the bare alkaloid aglycon, solanidine, characterized by the ion at *m*/*z* 398.3415.

## 4. Conclusions

In this research, for the first time, the extraction of 5-CQA from potato sprouts was in depth evaluated. With this aim, a UAE procedure was properly optimized through an Experimental Design. The results showed a negative impact of water content in ethanol and extraction time on the 5-CQA extraction efficiency, suggesting the economic and environmentally friendly character of the proposed method. Ascorbic acid used as additive to the extraction solvent proved to be an effective anti-browning agent. Moreover, the freeze-drying of potato sprouts also showed a positive role in controlling the enzymatic browning reaction and ensuring high concentration of bioactives. An optimized HPLC-DAD method was successfully applied to quantify and monitor the integrity of 5-CQA and its main isomers, as well as caffeic acid, while an HPLC-HRMS/MS investigation allowed us to confirm their identity, along with that of the two major glycoalkaloids. Furthermore, the optimized extraction conditions provided enhanced in vitro bioactivity of the final extract.

The outstanding results achieved from our investigation could pave the way for the exploitation of potato sprout wastes. On the one hand, the development of purification steps to separate phenols from glycoalkaloids and obtain ready-to-use CQA extracts in the food industry would be highly desirable; on the other hand, the assessment of the multifaceted aspects of the potential role of glycoalkaloids as anticancer by inducing apoptosis and inhibiting cell growth could turn the tide from “burden to blessing” compounds.

## Figures and Tables

**Table 1:** Extraction yield (%), TPC and in vitro antioxidant activity (measured with the FRAP, DPPH, and ABTS assays) of sprout extracts obtained by ME and UAE. Results, reported as mean value ± standard deviation (*n* = 3), are expressed on dry weight *.

Extraction Technique	Yield (%)	TPC (mg GAE ^†^/g)	FRAP (mg TE ^‡^/g)	DPPH (mg TE ^‡^/g)	ABTS (mg TE ^‡^/g)
ME	22.77 ± 0.08 ^a^	0.70 ± 0.07 ^a^	8.57 ± 0.07 ^a^	6.60 ± 0.66 ^A^	9.38 ± 0.11 ^a^
UAE	21.32 ± 0.07 ^b^	1.55 ± 0.09 ^b^	10.17 ± 0.21 ^b^	8.04 ± 0.53 ^B^	8.55 ± 0.22 ^b^

* based on 70% water loss. ^†^ GAE: gallic acid equivalents. ^‡^ TE: trolox equivalents. Different lowercase and uppercase letters in each column indicate significant differences with *p*-value lower than 0.01 and 0.05, respectively.

**Table 2:** Comparison of the phenolic acid content between fresh and oven-dried sprout samples. Results, reported as mean value ± standard deviation (*n* = 3), are expressed on dry weight.

Samples	3-CQA (μg/g)	CA (μg/g)	5-CQA (μg/g)	4-CQA (μg/g)
Fresh sprouts	9.86 ± 0.32	68.09 ± 0.29 ^a^	227.08 ± 6.90 ^a^	15.33 ± 1.50
40 °C-dried sprouts	-	43.31 ± 2.13 ^b^	23.39 ± 0.58 ^b^	-
70 °C-dried sprouts	-	11.91 ± 0.11 ^c^	37.05 ± 1.11 ^c^	-

Different lowercase letters within the same column indicate significant differences with *p*-value lower than 0.01.

**Table 3:** Comparison of the phenolic acid content in fresh sprout samples treated with three different extraction solvents. Results are expressed as mean value ± standard deviation (*n* = 3).

Extraction Solvent System	3-CQA (μg/g)	CA (μg/g)	5-CQA (μg/g)	4-CQA (μg/g)
Acetone/water (70:30, *v*/*v*) + HAc 1.7 mM	0.73 ± 0.00 ^a^	24.81 ± 3.04 ^a^	22.67 ± 1.87 ^a^	3.58 ± 0.64 ^a,A^
Ethanol/water (70:30, *v*/*v*) + HAc 1.7 mM	3.35 ± 0.17 ^b^	31.83 ± 0.17 ^b^	51.56 ± 0.39 ^b^	3.08 ± 0.54 ^a,b,A,B^
Water (100%) + HAc 1.7 mM	1.19 ± 0.00 ^c^	6.11 ± 0.14 ^c^	11.39 ± 0.84 ^c^	2.24 ± 0.28 ^b,B^

Different lowercase and uppercase letters in each column indicate significant differences with *p*-value lower than 0.01 and 0.05, respectively.

**Table 4:** Phenolic acid content (μg/g) in a fresh sprout sample extracted according to the conditions of Experimental Design.

Experiment	3-CQA (μg/g)	CA (μg/g)	5-CQA (μg/g)	4-CQA (μg/g)
N1	13.3	51.41	566.43	25.38
N2	0.13	7.91	34.53	4.66
N3	8.66	49.47	333.79	20.37
N4	3.41	22.45	110.68	16.77
N5	3.19	13.53	141.86	5.19
N6	0.80	7.85	24.09	4.21
N7	1.41	30.33	172.51	5.06
N8	1.40	16.06	99.57	15.05
N9	24.84	16.44	273.65	28.83
N10	26.04	35.32	230.92	16.75
N11	25.44	25.88	252.28	22.79

**Table 5:** Phenolic acid content (μg/g) in a fresh sprout sample extracted with different acid additives (HAc, CitA and AsA) at two different concentrations (1.7 μM and 1.7 mM). Results are expressed as mean value ± standard deviation (*n* = 3).

Acid Additive	Conc.	3-CQA (μg/g)	CA (μg/g)	5-CQA (μg/g)	4-CQA (μg/g)
HAc	1.7 µM	16.32 ± 0.42 ^a^	23.67 ± 0.08 ^a^	511.62 ± 9.29 ^a,A^	88.66 ± 0.76 ^a^
CitA		10.92 ± 0.07 ^b^	22.78 ± 0.09 ^b^	349.73 ± 2.62 ^b,B^	63.96 ± 1.76 ^b^
AsA		17.79 ± 0.61 ^c^	25.98 ± 0.06 ^c^	491.25 ± 9.84 ^a,C^	99.79 ± 0.83 ^c^
HAc	1.7 mM	41.27 ± 3.57 ^a^	40.06 ± 0.01 ^a,A^	1468.20 ± 46.82 ^a^	261.60 ± 8.60 ^a,A^
CitA		53.83 ± 4.52 ^b^	74.87 ± 12.86 ^a,b,B^	1766.71 ± 23.81 ^b^	240.74 ± 5.42 ^a,A^
AsA		140.58 ± 2.36 ^c^	91.13 ± 3.98 ^b,C^	3158.71 ± 50.29 ^c^	514.26 ± 18.18 ^b,B^

Different lowercase and uppercase letters in each column indicate significant differences with *p*-value lower than 0.01 and 0.05, respectively.

**Table 6:** Phenolic acid content (μg/g) in a freeze-dried sprout sample extracted with different acid additives at two different concentrations (1.7 μM and 1.7 mM) (Stage-2 sprouts). The bottom row refers to freeze-dried Stage-3 sprouts (acid additive 1.7 mM). Results are expressed as mean value ± standard deviation (*n* = 3).

Sprouting Time	Acid Additive	Conc.	3-CQA (μg/g)	CA (μg/g)	5-CQA (μg/g)	4-CQA (μg/g)
2 months	HAc	1.7 µM	186.33 ± 1.33 ^A^	399.76 ± 15.21 ^A^	3840.41 ± 146.10 ^A^	642.62 ± 2.79 ^a^
	CitA		144.23 ± 6.33 ^B^	399.01 ± 56.60 ^A^	3041.53 ± 36.40 ^B^	615.45 ± 5.70 ^b^
	AsA		186.43 ± 6.95 ^A^	300.96 ± 5.52 ^B^	3778.05 ± 72.51 ^A^	468.38 ± 2.88 ^c^
	HAc	1.7 mM	193.46 ± 15.00 ^A^	256.36 ± 19.06 ^A^	4795.62 ± 22.95 ^a,b,A^	62.01 ± 7.43 ^a^
	CitA		150.45 ± 6.12 ^B^	247.63 ± 6.37 ^A^	4211.25 ± 276.04 ^a,B^	685.94 ± 13.98 ^b^
	AsA		186.73 ± 6.10 ^A^	80.68 ± 0.66 ^B^	4980.05 ± 287.15 ^b,A^	788.00 ± 5.00 ^c^
5 months	HAc	1.7 mM	194.02 ± 3.69 ^a^	44.27 ± 0.41 ^A^	1621.54 ± 31.78 ^a^	341.70 ± 2.72 ^a^
	AsA		269.13 ± 6.32 ^b^	39.98 ± 4.94 ^A^	1207.07 ± 124.83 ^b^	433.47 ± 8.75 ^b^

Different lowercase and uppercase letters in each column indicate significant differences with *p*-value lower than 0.01 and 0.05, respectively.

## Data Availability

The data presented in this study are available in this manuscript.

## Supplementary Materials

The following supporting information can be downloaded at: https://www.mdpi.com/article/10.3390/antiox12020348/s1, Figure S1: HPLC-DAD profiles of (A) a standard mixture of CA, 5-CQA and its isomers 3- and 4-CQA; (B) a fresh potato sprout sample and two oven-dried samples at (C) 40 °C and (D) 70 °C; Figure S2: Chromatographic profile of CQAs and CA from potato sprouts obtained in the optimal extraction conditions [freeze-dried sample; extraction solvent water/ethanol-70:30 (*v*/*v*) containing AsA 1.7 mM; solid/solvent ratio 1:10 (g/mL); UAE time 5 min]; Figure S3: (A) Full-scan EIC chromatograms of CQA isomers obtained from the analysis of the standards and of the real sample extract (L1); (B) MS^2^ spectra of 3-CQA (neochlorogenic acid), 5-CQA (chlorogenic acid), and 4-CQA (cryptochlorogenic acid) in the extract.; Figure S4: (A) Full-scan EIC chromatograms of CA (caffeic acid) and (B) MS^2^ spectra of CA obtained from the analysis of the standards and of the real sample extract (L1); Figure S5: MS^2^ spectra of (A) Ferulic acid, (B) Protocatechuic acid, (C) Coumaric acid and (D) Quercetin obtained from the analysis of the standards and of the real sample extract (L1 or L2); Figure S6: (A) Full-scan EIC chromatogram of α-solanine obtained injecting real sample extract (L2); (B) MS^2^ spectrum with the assignment of fragment ions obtained by using software package Mass Frontier^TM^; Figure S7: (A) Full-scan EIC chromatogram of α-chaconine obtained injecting real sample extract (L2); (B) MS^2^ spectrum with the assignment of fragment ions obtained by using software package Mass Frontier^TM^; Table S1: Calibration data: regression equation, linearity range, coefficient of determination value (R^2^), LOD and LOQ values; Table S2: Method validation: evaluation of precision (RSD %) and accuracy (Recovery %) in the short- and long-term period (intra-day and inter-day precision and accuracy).
