# Supplementary material for: Optimization of Ultrasound-Assisted Extraction of Chlorogenic Acid from Potato Sprout Waste and Enhancement of the In Vitro Total Antioxidant Capacity
Source: Antioxidants (Basel). 2023 Feb 1;12(2):348. doi: 10.3390/antiox12020348 (PMC9952679; doi:10.3390/antiox12020348)
Supplement: Supplementary file 1 [file antioxidants-12-00348-s001.zip › antioxidants-2146714-supplementary.pdf]

antioxidants

Supplementary Material

Optimization of Ultrasound-Assisted Extraction of Chlorogenic Acid from Potato Sprout Waste and Enhancement of the *in vitro* Total Antioxidant Capacity

Luciano Mangiapelo [1,†], Francesca Blasi [1,†], Federica Ianni [1,*], Carolina Barola [2], Roberta Galarini [2], Ghaid WA Abualzulof [1], Roccaldo Sardella [1,3], Claudia Volpi [4], Lina Cossignani [1,3]

[1] Department of Pharmaceutical Sciences, University of Perugia, Via Fabretti 48, 06123 Perugia, Italy
[2] Istituto Zooprofilattico Sperimentale dell'Umbria e delle Marche "Togo Rosati", 06126 Perugia, Italy
[3] Center for Perinatal and Reproductive Medicine, Santa Maria della Misericordia University Hospital, University of Perugia, Sant'Andrea delle Fratte, 06132 Perugia, Italy
[4] Department of Medicine and Surgery, University of Perugia, Piazzale Gambuli 1, 06132 Perugia, Italy
* Correspondence: federica.ianni@unipg.it; Tel.: +39-075-585-7955
† These authors contributed equally to this work

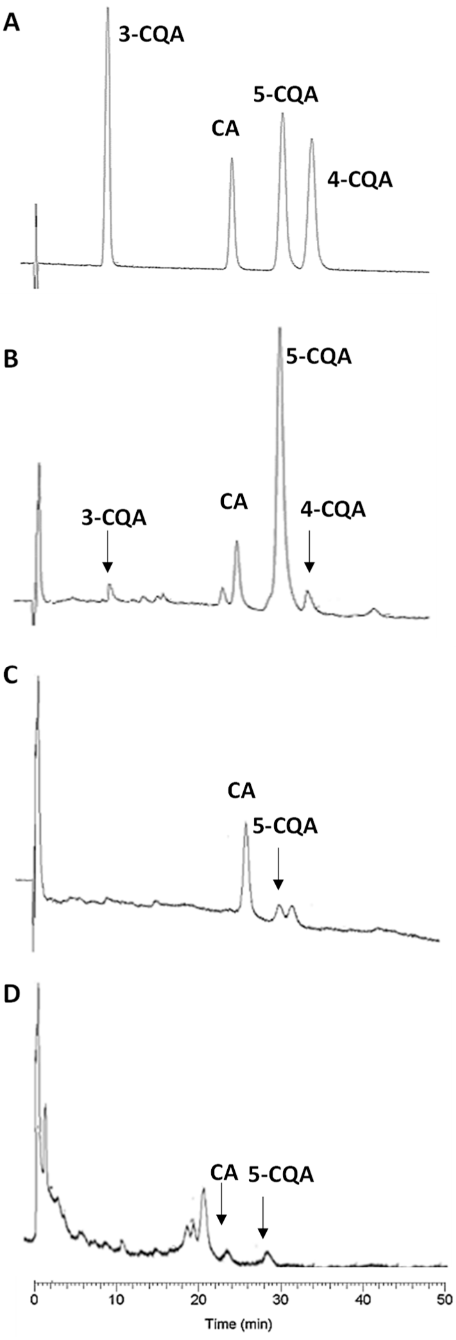

Figure S1. HPLC-DAD profiles of A) a standard mixture of CA, 5-CQA and its isomers 3- and 4-CQA; B) a fresh potato sprout sample and two oven-dried samples at C) 40 °C and D) 70 °C.

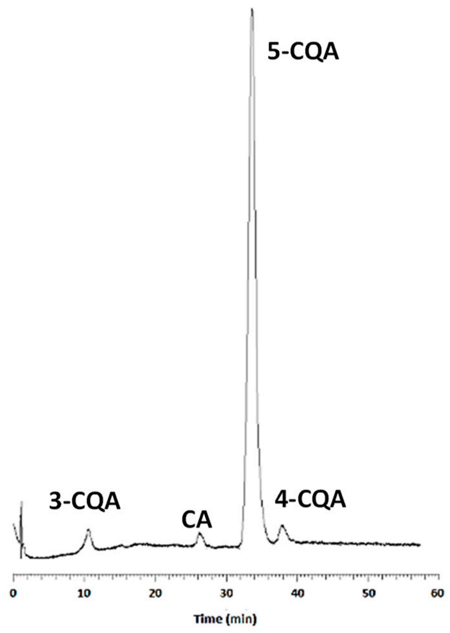

Figure S2. Chromatographic profile of CQAs and CA from potato sprouts obtained in the optimal extraction conditions [freeze-dried sample; extraction solvent water/ethanol-70:30 (v/v) containing AsA 1.7 mM; solid/solvent ratio 1:10 (g/mL); UAE time 5 min].

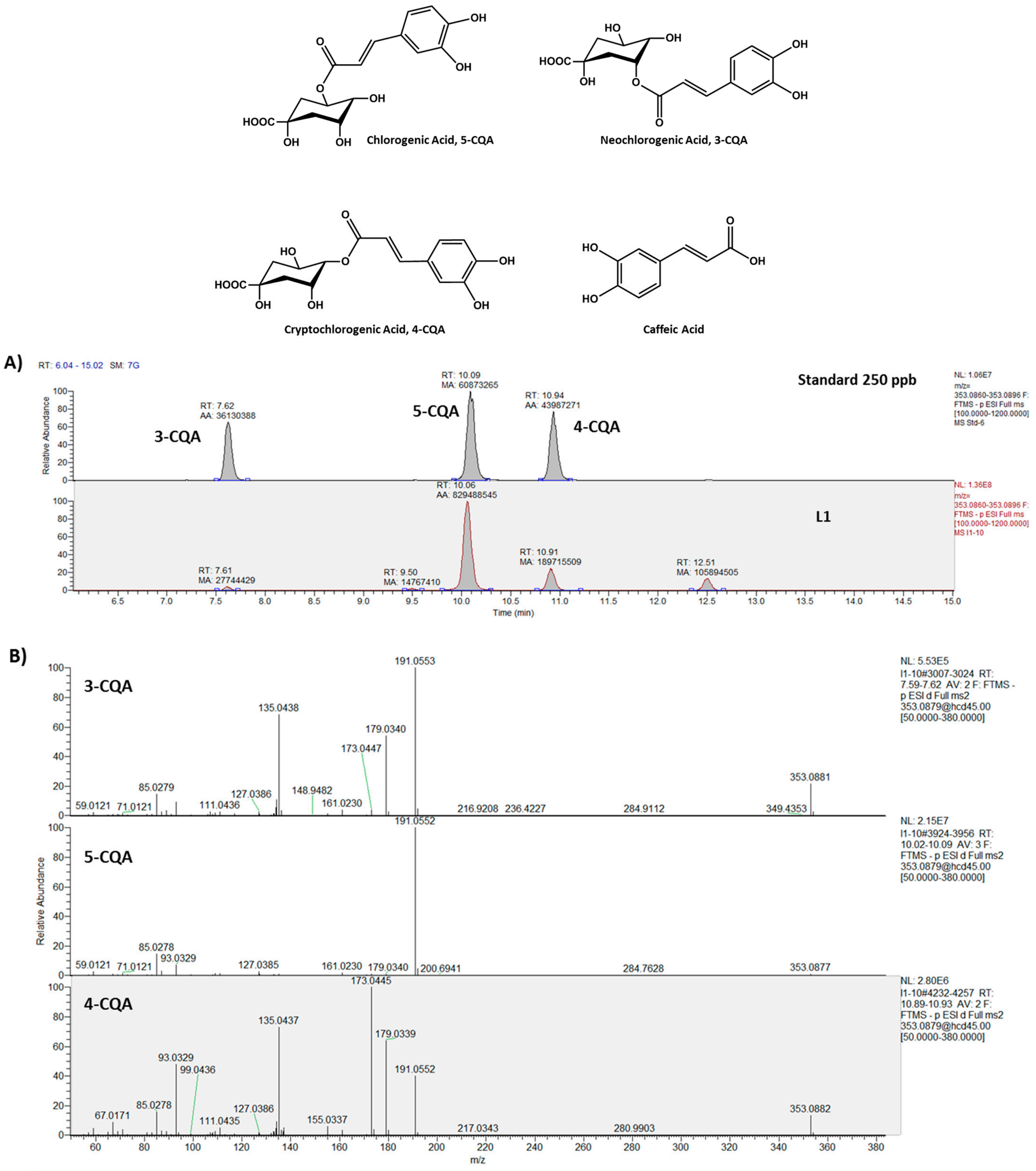

Figure S3. A) Full scan EIC chromatograms of CQA isomers obtained from the analysis of the standards and of the real sample extract (L1); B) MS^2 spectra of 3-CQA (neochlorogenic acid), 5-CQA (chlorogenic acid), and 4-CQA (cryptochlorogenic acid) in the extract.

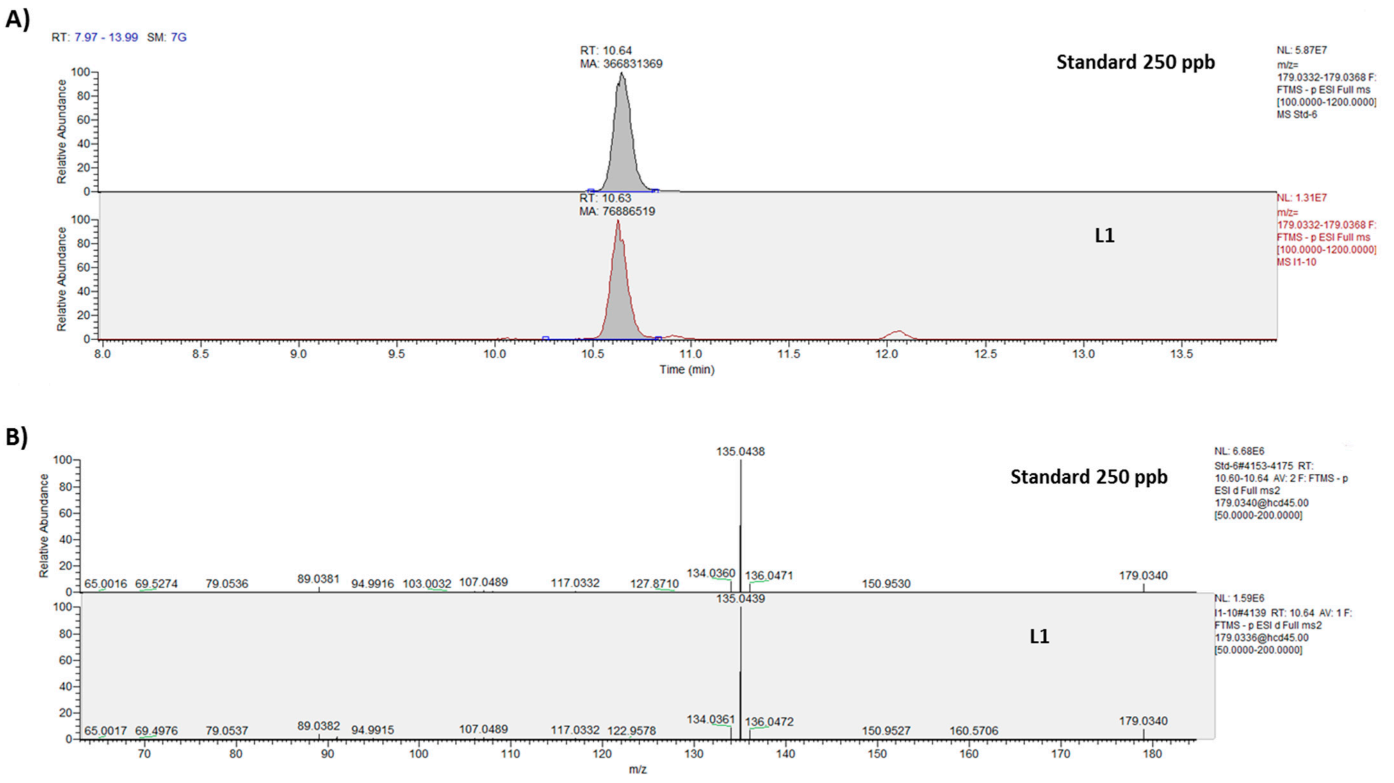

Figure S4. A) Full scan EIC chromatograms of CA (caffeic acid) and B) MS^2 spectra of CA obtained from the analysis of the standards and of the real sample extract (L1).

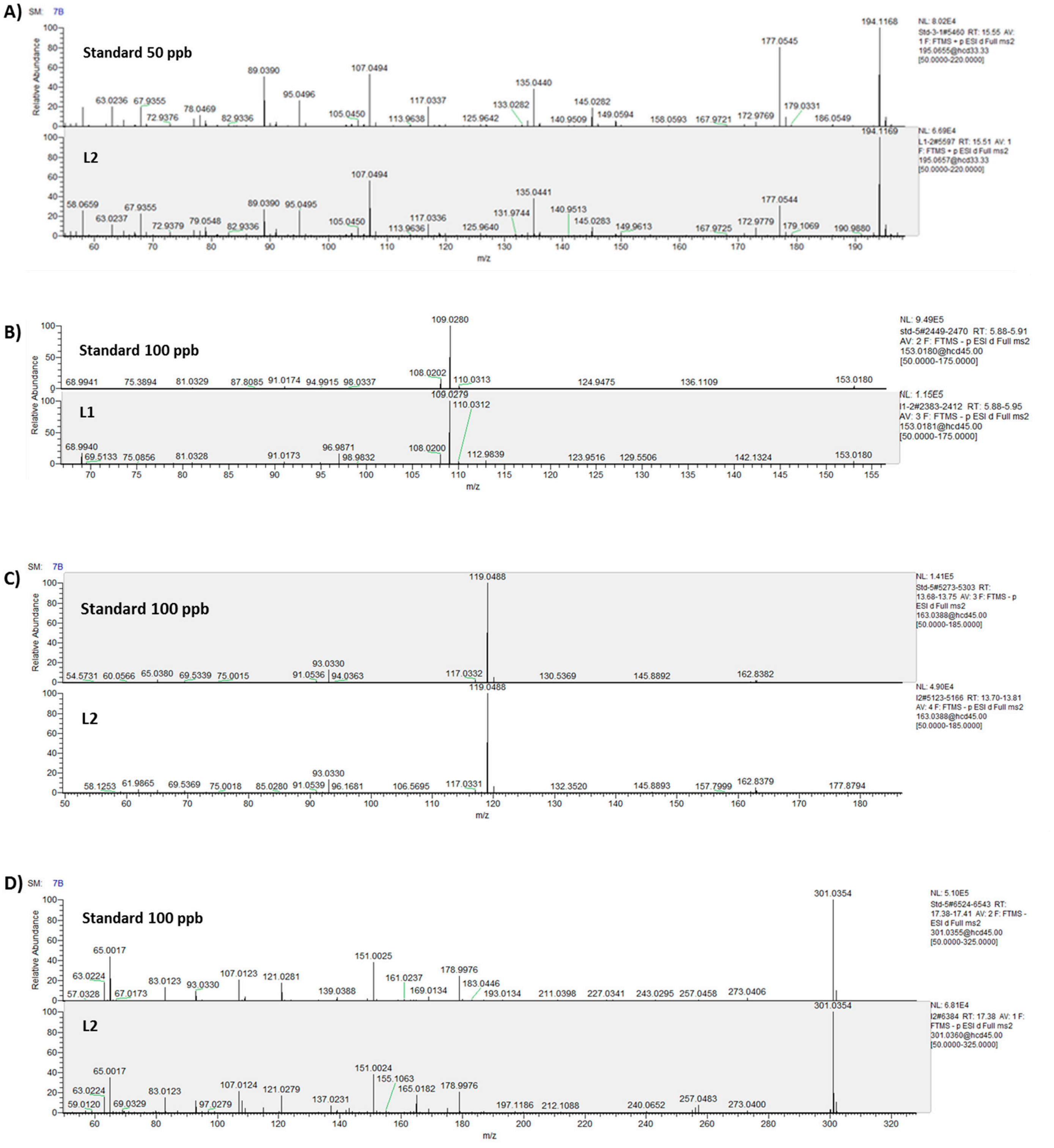

Figure S5. MS^2 spectra of A) Ferulic acid, B) Protocatechuic acid, C) Coumaric acid and D) Quercetin obtained from the analysis of the standards and of the real sample extract (L1 or L2).

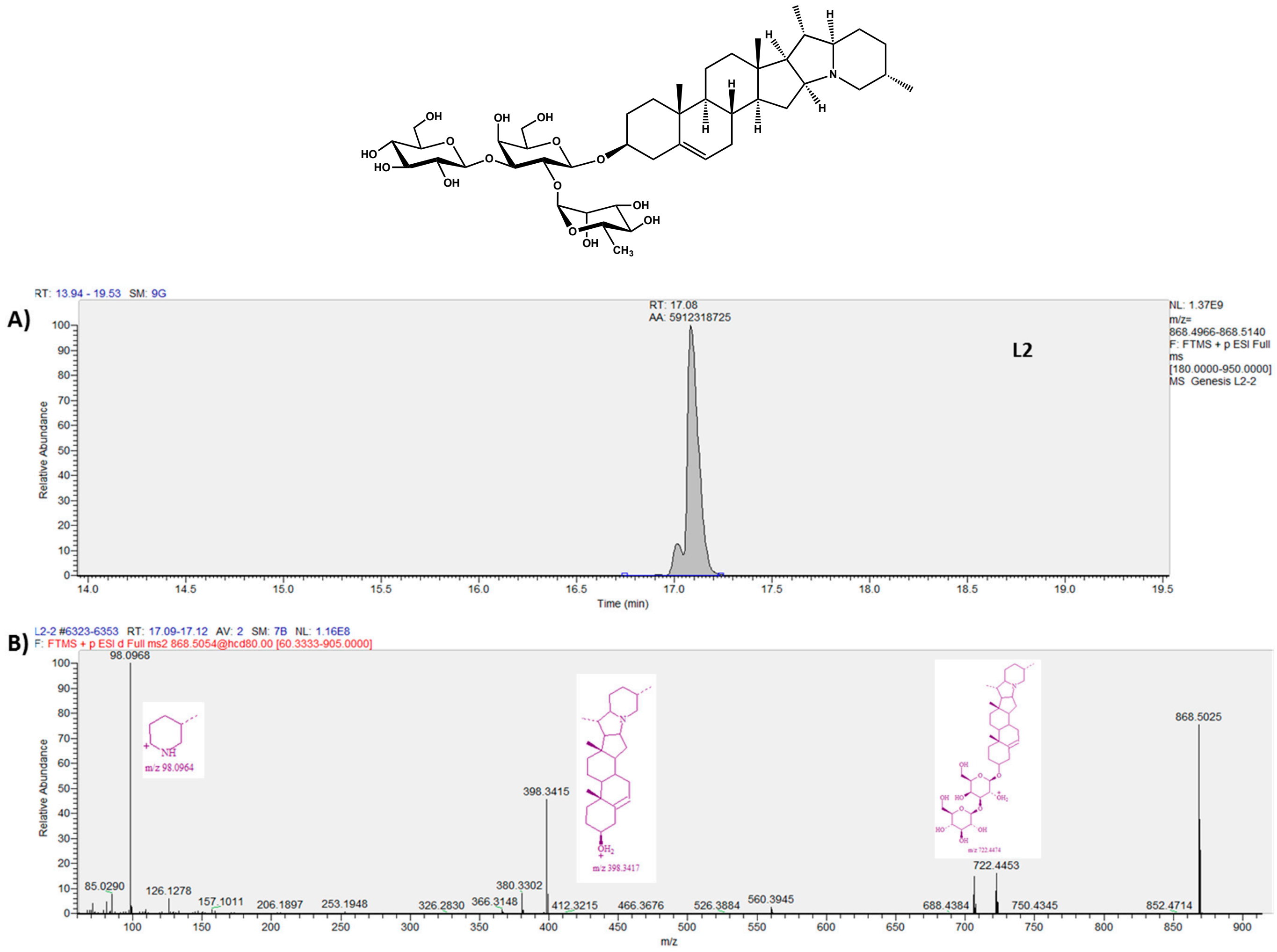

Figure S6. A) Full scan EIC chromatogram of α-solanine obtained injecting real sample extract (L2); B) MS[2] spectrum with the assignment of fragment ions obtained by using software package Mass Frontier™.

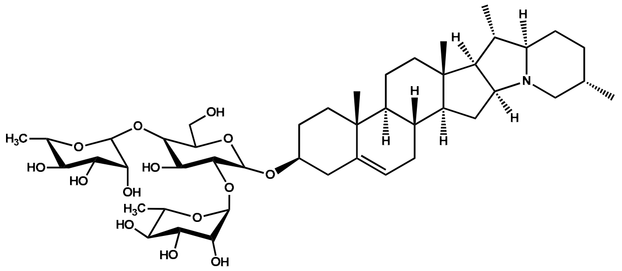

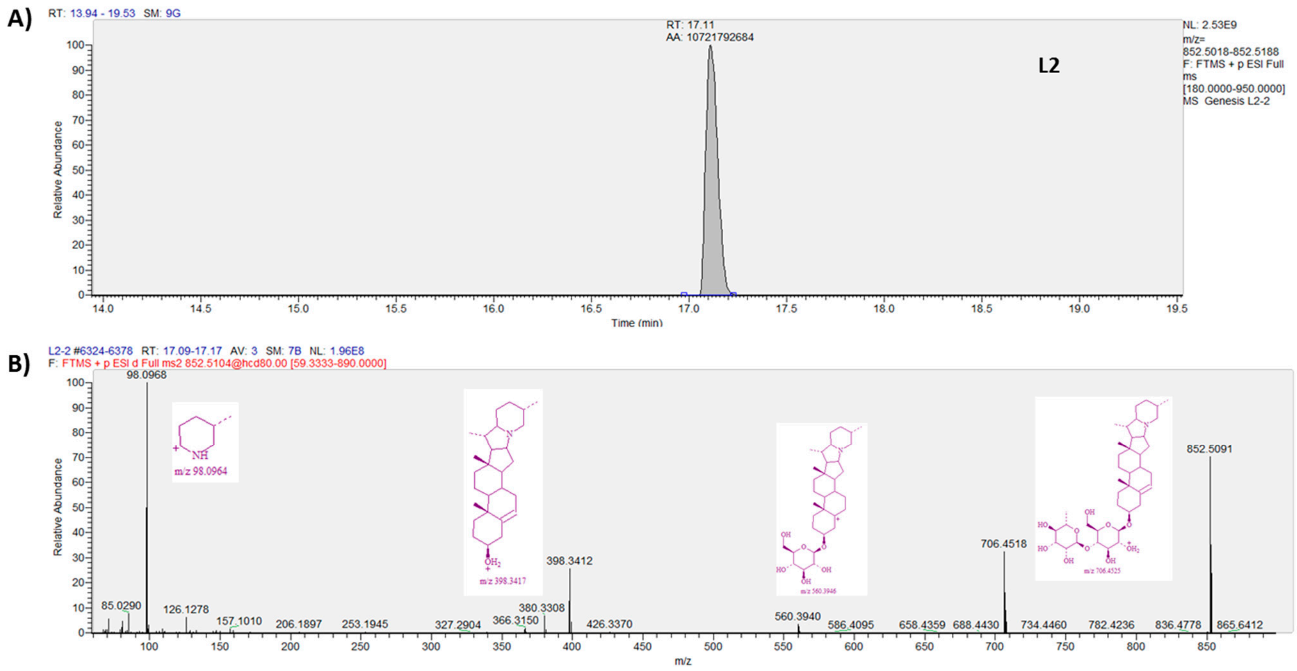

Figure S7. A) Full scan EIC chromatogram of α-chaconine obtained injecting real sample extract (L2); B) MS² spectrum with the assignment of fragment ions obtained by using software package Mass Frontier™.

Table S1. Calibration data: regression equation, linearity range, coefficient of determination value (R^2), LOD and LOQ values.

Cpd	Regression Equation	Linearity range (µg/mL)	R^2	LOD* (ng/mL)	LOQ* (ng/mL)
**3-CQA**	y = 63669.27(±360.52)x + 410.03(±176.60)	0.04-22	0.9998	9.1	27.7
**CA**	y = 30196.86(±386.64)x - 397.73(±77.49)	0.04-22	0.9988	8.5	25.7
**5-CQA**	y = 61299.27(±1060.21)x - 8143.80(±57.82)	0.04-22	0.9980	3.1	9.4
**4-CQA**	y = 54442.76(±476.30)x - 3569.87 (±114.30)	0.04-22	0.9995	6.9	20.9

*LOD and LOQ values were calculated as the standard deviation of the response (s_y) on the slope of the calibration curve (b), according to the equations: C_{LOD}= 3.3(s_y/b) and C_{LOQ} = 10(s_y/b).

Table S2. Method validation: evaluation of precision (RSD %) and accuracy (Recovery %) in the short- and long-term period (intra-day and inter-day precision and accuracy).

Nominal conc. (µg/mL)	Intra-day mean concentration (µg/mL)				Intra-day Precision (RSD%)				Intra-day Accuracy (Recovery%)			
	3-CQA	CA	5-CQA	4-CQA	3-CQA	CA	5-CQA	4-CQA	3-CQA	CA	5-CQA	4-CQA
3.65	3.65	3.68	3.79	3.78	0.69	0.35	1.18	0.28	100	101	104	104
	3.68	3.63	3.75	3.84	0.38	7.18	3.02	2.83	101	99.5	103	105
	3.68	3.85	3.82	3.80	0.46	0.56	0.33	0.65	101	106	105	104
14.6	14.2	14.7	14.8	14.8	0.23	1.23	0.86	0.60	97.5	101	102	101
	14.3	14.2	14.3	14.8	0.32	3.45	2.72	0.53	97.8	97.0	97.7	101
	14.3	14.0	14.3	14.8	0.45	5.58	2.64	0.23	97.6	95.9	98.1	101

Nominal conc. (µg/mL)	Inter-day mean concentration (µg/mL)				Inter-day mean Precision (RSD%)				Inter-day Accuracy (Recovery%)			
	3-CQA	CA	5-CQA	4-CQA	3-CQA	CA	5-CQA	4-CQA	3-CQA	CA	5-CQA	4-CQA
3.65	3.67	3.72	3.81	3.81	0.61	4.42	1.82	1.62	101	102	104	104
14.6	14.3	14.3	14.5	14.8	0.33	4.02	2.65	0.42	97.6	97.9	99.1	101
